# Financing the national health insurance in South Africa to achieve an appropriate degree of coverage of effective health services and levels of financial protection - a descriptive, experimental quantitative study

**DOI:** 10.11604/pamj.2025.52.66.44779

**Published:** 2025-10-09

**Authors:** Samuel Vuyo Mokoena, Panjasaram Naidoo

**Affiliations:** 1 University of KwaZulu-Natal, School of Health Sciences, Westville Campus, South Africa

**Keywords:** Healthcare, reform, risk-pooling, funding, insurance

## Abstract

**Introduction:**

the financial obligations that come with the implementation of a system that will ensure equitable access to healthcare are an important consideration to realise the national health insurance (NHI). The aim of this manuscript was to evaluate whether there is sufficient policy consensus in finding innovative and sustainable ways to address the healthcare finance fundamentals of the NHI.

**Methods:**

a descriptive, experimental quantitative study employing a 5-point Likert scale questionnaire was used to collect the data. The participants were drawn from individuals and stakeholders who interact with and/or are in the employment of the organisations concerned with healthcare regulation and service delivery.

**Results:**

the study found that the funding initiatives employing budget prioritization for healthcare reforms (76%), reducing reliance on payroll taxes (68%), and permitting the private insurance to play a role (62%) were preferred; however, 70% of the respondents were opposed to new payroll taxes. The study found that 64% endorsed supply-side policies for benefit package expansion, 60% supported capping health expenditure and restricting catastrophic co-payments, and 77% favoured prioritizing primary care and cost-effective services. There was limited support (42%) for the consolidation of medical aids, and opinions were divided on maintaining multiple risk pools. There was consensus across occupational groups on NHI funding (P = 0.603) but marked differences by occupation for "getting more value for money" (P = 0.041) and "pooling and redistribution of resources" (P = 0.032), particularly between leadership/governance and information/research-service delivery roles. Correlation analysis showed the three main subthemes had moderate positive relationships (r = 0.34-0.66), suggesting that challenges or improvements in one area are likely to impact others.

**Conclusion:**

these findings highlight a critical need for nuanced, occupation-sensitive approaches to policy development and implementation, given the fact that there are divergent views on funding policies.

## Introduction

The NHI scheme´s policy in South Africa is fraught with many challenges, which threaten its feasibility, financial apportionment being one of them. Critics have constantly questioned the feasibility and rationale of some of the options proposed for the funding of the NHI, as seen in the findings of a 2019 report by Van den Heever *et al*. as well as an article responses and options for the Portfolio Committee on Health, which in a nutshell called for revision and fresh consultation on the NHI Bill [1,2]. The 2020 South African healthcare expenditure, according to the World Health Organization, retrieved on 7^th^ April, 2023, indicated a current health expenditure of 8.58% of the South African gross domestic product (GDP) [3]. The NHI white paper recorded that the estimates that were published in the NHI green paper (R256 billion in 2010 terms) will take public health spending from around 4% to 6.2% of GDP by 2025/2026, assuming a growth of 3.5% in the economy [4]. The above scenario lays down the financial situation under which the financing options for the NHI are supposed to play out. The NHI white paper (2017) details three approaches that will be employed to finance the NHI, those being: various tax options, pooling of revenues, and cost-containment and management improvement measures [4].

Despite the fact that the National Development Plan (NDP) 2030 sets out clear objectives to achieve the NHI, the challenges pertaining to financing, the NHI lingers on [5]. These objectives are encapsulated in the four pre-requisites: improving the quality of public health care, lowering the relative cost of private care, recruiting more professionals in both the public and private sectors, and developing a health information system that spans public and private health providers [5]. This paper evaluates how the government´s intention of improving the quality of public health care and lowering the relative cost of private care can be supported and enhanced. The paper seeks to establish if the healthcare financing policies will: (i) deal sufficiently with the fundraising avenues and expand as well as sustain coverage; (ii) ensure more value for the money spent; and (iii) effective pooling and redistribution of resources to ensure equity and financial protection.

Arguably, South Africa´s situation when it comes to this point is not unique, as a number of countries, including those in the Asia-Pacific, as alluded to in other studies, have faced challenges in finding the fiscal space to finance UHC policies and programs since coverage expansion calls for a huge increase in public spending [6]. According to Yeoh *et al*. (2019), these countries embarked on a number of policy approaches unique to their situations to deal with these challenges [6] - which aligns with the findings of a review study by Debie *et al*.(2022) that concluded context-specific health policy and health financing modalities helped to speed up progress towards universal health coverage (UHC) and health security [7]. For instance, in raising funds to expand and sustain coverage, the Asia-Pacific countries have seen those countries prioritising health budget alongside macroeconomic growth, diversifying sources of revenue, and some (low-income countries such as Bangladesh and Ethiopia) seeking ways to expand their narrow tax basis by introducing new payroll taxes [6].

This study aimed to evaluate whether there is sufficient policy consensus in finding innovative and sustainable ways to address the healthcare finance fundamentals of the NHI.

## Methods

**Study design and setting:** the study was undertaken as a descriptive, experimental, quantitative study, and it employed the use of a questionnaire to collect the data. The target population was drawn from people involved in leadership and governance in the areas of healthcare funding and management, medical products and technologies, and information and research in healthcare in South Africa.

**Study population:** individuals and stakeholders who interact with and/or are in the employment of statutory health councils, regulatory authorities, medical schemes administrators, medical schemes, and voluntary professional organisations were invited to participate in the study. Health care workers registered with the South African Pharmacy Council (SAPC), South African Nursing Council (SANC), and Health Professions Council of South Africa (HPCSA) were also invited as participants. They were selected on the basis that they are important stakeholders with a keen interest in contributing meaningfully to the NHI discourse due to the wealth of experience in health care delivery. The participants were seen to be in the forefront of the healthcare industry engagements aimed at improving healthcare, with a keen interest in the developments around NHI, and have the capacity to influence sentiment around the attainment of the NHI objectives. The study excluded participants who were not in the healthcare sector.

A sample of 660 participants was determined to be adequate for the study. Given α=0.05, β=0.2, and df = 65, and using GPower 3.1.9.7 as inputs in the sample size calculation software, it was estimated that a minimum sample size of 660 was required to detect small to medium effect sizes of at least 0.25 about 80% of the time (have 80% power of test) with a 95% confidence.

**Data collection tool:** the tool was a 5-point Likert scale web-based anonymous survey questionnaire that was distributed via SurveyMonkey™ over a period of at least two months from 25^th^ October to 6^th^ December 2021. The survey questionnaire was designed to collect the population demographics, as well as responses to questions related to policy approaches that addressed challenges that affect healthcare financing functions in the implementation of the NHI.

**Statistical analysis:** the data analysis was conducted using the R Statistical computing software of the R Core Team, 2020, version 3.6.3. The results were presented in the form of descriptive and inferential statistics. Where applicable, the descriptive statistics of numerical measurements were summarised as the minimum, maximum, quartiles, interquartile range, means, standard deviation, and coefficient of variation. On the other hand, the categorical variables were described as counts and percentage frequencies. Some of the results were visualised in the form of bar charts, Likert, and correlation plots. Cronbach's alpha was used to measure the internal consistency of items that were grouped into a section (construct). Kruskal-Wallis was applicable in testing differences in the scores between at least three groups, followed by the Wilcox pairwise tests. All the statistical tests were conducted at a 5% level of significance.

**Ethical considerations:** approval for the research study was obtained from the University of Kwa-Zulu Natal´s Humanities and Social Sciences Research Ethics Committee (HSSREC) with ethics number: HSSREC 00002565/2021. Informed consent was obtained from the participants prior to data collection.

## Results

**General characteristics of the study population:** a total of 678 responses were received, exceeding the calculated sample size of 660, giving a response rate of 102%. The respondents were deployed in both the public and private healthcare sectors. The responses received were from the health workforce (67%), followed by medical products and technologies (13.3%), and 1.3% of the responses received from NHI implementers.

**Figure 1:**
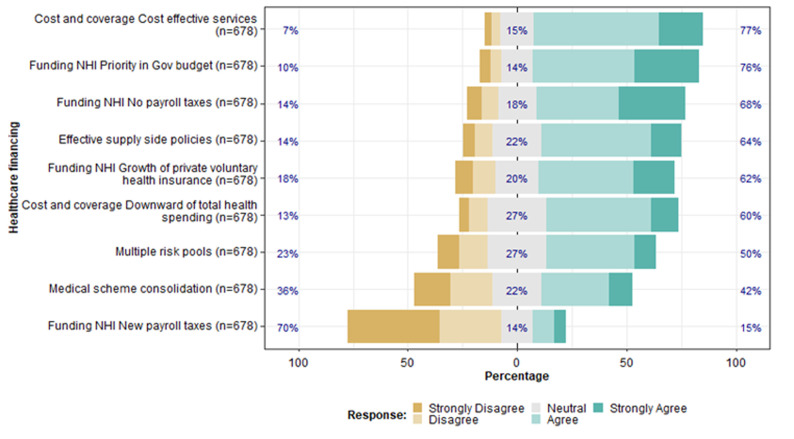
participants' responses for the healthcare financing theme (n=678)

**Participants´ response per subtheme:** the healthcare financing theme explored the challenges inherent with raising revenues to expand and sustain coverage, getting value for money, and managing effective pooling and redistribution of resources to ensure equity and financial protection. The results are presented as subthemes and are further analysed by occupation and correlation between the subthemes (Figure 1). The internal consistency for the healthcare financing theme was high, with a Cronbach's Alpha of 0.821, an indication that the items reliably measured the intended concepts.

**Funding the NHI (n=678):** most participants (76%) are in favour of the government raising revenues to fund the NHI from budget prioritisation alongside macro-economic growth. Sixty-eight percent of participants favoured reducing the overreliance on payroll taxes. Sixty-two percent favoured efforts to permit the growth of private voluntary health insurance to supplement statutory coverage. However, 70% were totally opposed to the introduction of new payroll taxes.

**Getting more value for money (n=678):** majority of participants (64%) were in favour of effective supply-side policies to bring a balanced approach to prioritizing services and medicines for benefit package expansion, strong negotiation with pharmaceutical companies, and leveraging provider payment systems. Sixty percent supported a total cap on health expenditure and restriction of catastrophic co-payments in order to provide financial protection. An overwhelming majority (77%) of the participants favoured the utilization of more cost-effective services, such as emphasizing primary care in the benefits package and encouraging investment in facilities providing high-priority services, as ways of getting more value for money.

**Pooling and redistribution of resources (n=678):** the support for consolidation of medical schemes to improve cross-plan fairness and achieve integration and cross-subsidization is limited, as only 42% supported the idea, with 22% neutral and 36% opposed to the idea. Similarly, the participants are divided as to whether the transition to NHI could be supported by the maintenance of multiple risk pools. The study found that while 50% support the idea, 27% are neutral, with 23% opposed to the idea.

**Participants' reactions to NHI healthcare financing initiatives by occupation:** the occupation “other” was excluded from the statistical inference involving comparison of the scores between the groups, as it had only two participants, resulting in n=676. Though some of the subthemes of healthcare financing show differences in the responses by occupation based on the P-values as shown in Table 1, interestingly all the occupation groups seem to hold the same view when it comes to the subtheme funding the NHI, with a P-value of 0.603, where a P-value < 0.05 would indicate that there was a significant difference in the responses between the different occupations.

**Table 1:** healthcare financing subthemes per occupation

Occupation	Health workforce	Healthcare financing	Leadership and governance	Medical products and technologies	Other - academia	Implementer of the NHI	Information and research - service delivery	P-value
	(n=454)	(n=10)	(n=73)	(n=90)	(n=18)	(n=9)	(n=22)	
**Funding the NHI**								Kruskal
Mean ± sd (cv%)		68.5±12.5(18.2)	69.8±12.0(17.2)		65.3±13.8(21.1)	67.2±16.8(25.0)	65.0±14.6(22.4)	
Median(q1-q3)	65.0(60.0-75.0)	70.0(61.3-78.8)	70.0(60.0-80.0)	70.0(60.0-75.0)	70.0(57.5-70.0)	65.0(60.0-70.0)	70.0(56.3-70.0)	0.603
N (min-max)	454(20.0-100)	10(45.0-85.0)	73(40.0-95.0)	90(20.0-100)	18(40.0-95.0)	9(45.0-100)	22(30.0-95.0)	
**Getting more value for money**								Kruskal
Mean ± SD (cv%)		72.7±14.9(20.5)				80.7±10.8(13.3)	67.0±15.3(22.9)	
Median (q1-q3)	73.3(66.7-80.0)	73.3(61.7-85.0)	80.0(73.3-86.7)	80.0(66.7-80.0)	73.3(68.3-78.3)	80.0(73.3-86.7)	73.3(61.7-73.3)	0.041
N (min-max)	454(20.0-100)	10(46.7-93.3)	73(40.0-100)	90(20.0-100)	18(33.3-100)	9(66.7-100)	22(26.7-93.3)	
**Pooling and redistribution of resources**								Kruskal
Mean ± SD (cv%)		66.0±21.2(32.1)			61.1±24.0(39.2)	80.0±11.2(14.0)	62.3±18.5(29.7)	
Median (q1-q3)	60.0(50.0-80.0)	70.0(62.5-80.0)	70.0(60.0-80.0)	70.0(42.5-80.0)	60.0(40.0 -77.5)	80.0(80.0-80.0)	60.0(52.5-77.5)	0.032
N (min-max)	454(20.0-100)	10(20.0-90.0)	73(20.0-100)	90(20.0-100)	18(20.0-100)	9(60.0-100)	22(20.0-100)	

NHI: national health insurance; SD: Standard Deviation

**Figure 2:**
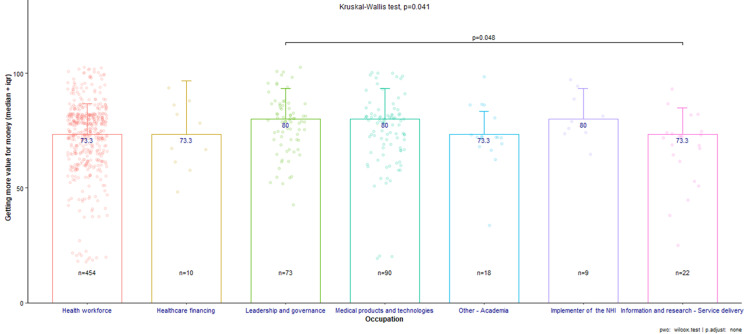
getting more value for money subthemes across all occupations

**Figure 3:**
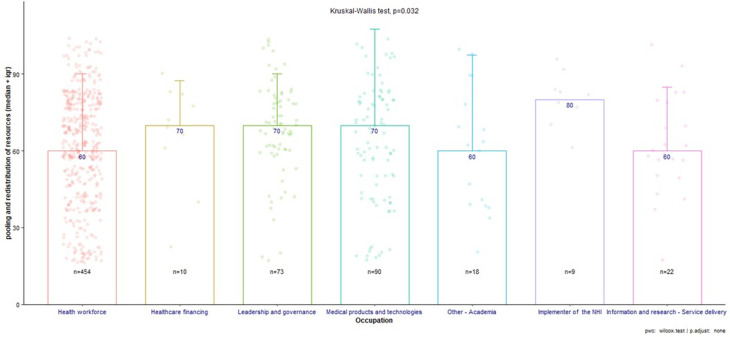
pooling and redistribution of resources subthemes across all occupations

There was no statistically significant difference for the subtheme "funding the NHI” between occupations (P-value = 0.603), indicating consensus across groups. However, there were significant differences in the subthemes "getting more value for money" (P-value = 0.041) and "managing effective pooling and redistribution of resources" (P-value = 0.032), as shown in Figure 2 and Figure 3. As per Figure 3the latter showed a statistically significant difference, particularly between those in leadership/governance and those in information/research-service delivery roles.

**Figure 4:**
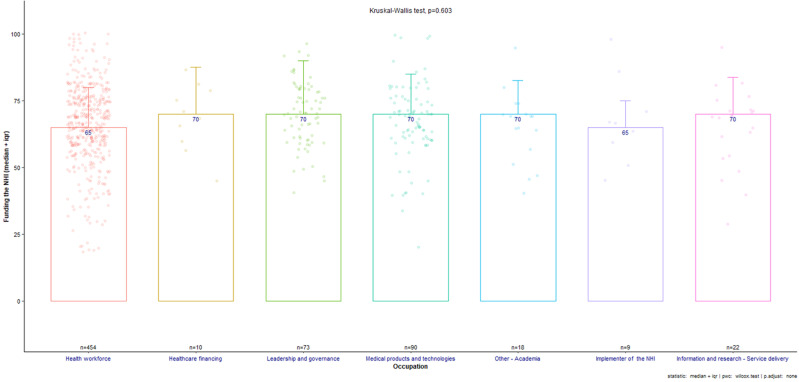
funding of the national health insurance subthemes across all occupations

The Kruskal-Wallis H test (Figure 4) indicated that there was no statistically significant difference in the ranking of responses from populations of different occupation groups in the “funding the NHI” subtheme, concurring with the findings above.

**Correlation of subthemes identified in healthcare financing:** correlation analyses (Table 2) were deployed to examine the relationships between the subthemes, using the following three cut-off points: 0-0.33 (weak correlation), 0.34-0.66 (moderate correlation), and 0.67-1.00 (strong correlation).

**Table 2:** correlation of the healthcare financing subthemes (P-value < 0.001)

	Variable 1	Variable 2	Correlation	P-value
1	Getting more value for money	Pooling and redistribution of resources	0.603	< 0.001
2	Funding the NHI	Getting more value for money	0.596	< 0.001
3	Funding the NHI	Pooling and redistribution of resources	0.582	< 0.001

NHI: national health insurance

The correlation analysis shows a positive monotonic relationship between the subthemes: as sentiment on one variable increased, so did sentiment on the other. All three pairs of themes identified in healthcare financing show a moderate positive correlation (0.34 to 0.66), an indication that improvements or challenges in one area of healthcare are likely to be reflected in the other areas.

## Discussion

The study assessed whether there was sufficient policy consensus in finding innovative and sustainable ways to address the healthcare finance fundamentals of the NHI in order to achieve the aspirations of UHC. Though there is consensus on all but three of the four approaches evaluated for the funding of the NHI, it is not clear if the NHI funding will follow these approaches.

The study established that there is a consensus on pursuing all funding sources, including expanding private voluntary health insurance (PVHI) alongside statutory coverage, to support universal health coverage (UHC) in line with South Africa´s 2030 vision for equitable health coverage [5]. Global studies reveal challenges in securing sufficient, sustainable government funding for UHC due to increased public spending needs [8]. Examples from Asia-Pacific countries show that using taxes on tobacco, alcohol, and sugar-sweetened beverages can effectively increase domestic health funding [9].

The study found that allowing the growth of PVHI as supplementary statutory coverage supports achieving the goals of South Africa's National Health Insurance (NHI). However, the 2019 NHI bill restricts medical schemes to only offer complementary coverage for services outside the NHI fund, potentially missing the benefits of PVHI's supplementary role [10]. Supporting this, previous studies highlight PVHI's important role in both developed and developing countries [11,12]. Additionally, a WHO technical brief emphasized that both private and public sectors are essential to health systems, with the private sector providing significant healthcare services in low- and middle-income countries [13].

South Africa must, however, guard against the unintended consequences of the growth of the private sector. A UNICEF study published in 2021 showed that in Bangladesh, India, and Pakistan, low public financing led to an overreliance on private providers for primary care without sufficient governance and regulation of costs and quality, leading to more than 78% of care being provided by private providers [14]. This inevitably led to an increase in out-of-pocket costs, leaving the marginalised society unprotected. The key messages in a WHO study on voluntary health insurance sum up the pros and cons of PVHI and its impact on UHC coverage and sustainability [15]. The WHO study states that it is difficult to attain universal health coverage by relying primarily on voluntary insurance scheme contributions [15].

The study found that the use of payroll taxes to fund the NHI was the least favoured option. Research points out that countries with a large informal sector, like Thailand, find it difficult to expand coverage through the introduction of payroll taxes; however, low-income countries such as Bangladesh and Ethiopia are still seeking to expand their narrow tax base by introducing new payroll taxes under their social insurance programme [8]. The implication of the finding is that South Africa needs to ensure that the unique circumstances of the country are taken into account when a decision on this policy matter is made.

The study found that the utilization of more cost-effective services, such as emphasizing PHC in the benefits package and encouraging investment in facilities providing high-priority services, is a way of getting more value for money. This finding is supported by a UNICEF study (2021) that states that UHC´s goals cannot be achieved without a focus on primary health care (PHC) components [14]. The study finding also aligns with both the 2011 UNICEF study and the UHC 2030 findings to invest in PHC [14,16-18].

The participants were in favour of effective supply-side policies to bring a balanced approach to prioritizing services and medicines for benefits package expansion, so as to bring more benefits to more people. The finding of the study is supported by the 2016 Lancet study, which asserts the use of these policy levers to increase efficiency to successfully manage costs without eroding coverage [7].

In terms of managing the effective pooling and redistribution of resources to ensure equity of financial protection, the study findings demonstrate that consolidation of medical schemes with a view to improving cross-plan fairness and achieving integration and cross-subsidization is not popular. Similarly, the participants were not convinced that the transition to NHI could be supported by the maintenance of multiple risk pools through a combination of standardized benefits and provider payments across plans to maintain equity in contributions and expenditures. On the face of it, these study findings presented a dichotomy on the issue of effective pooling and redistribution of resources.

A 2018 study by Chu *et al*. on Asia-Pacific countries highlighted a similar dual effect of integrating systems and merging risk pools [8,9]. Countries like Mongolia, the Philippines, and South Korea benefited from nationwide subsidies, uniform benefit packages, and lower administrative costs [9]. However, in Vietnam, this approach led to unintended negative effects where poorer rural areas ended up subsidizing wealthier urban provinces [9].

In Côte d´Ivoire, health reforms combined government and household funds to expand financial protection against disease through two schemes: a contributory general scheme and a non-contributory assistance scheme targeting the poorest [19]. This gradual pooling approach aimed to cover the entire population [19]. In summary, while integrating insurance schemes and pooling resources can improve fairness and efficiency in UHC, the outcomes depend heavily on country-specific contexts and can sometimes produce unintended inequities.

The studies emphasize that before making policy decisions on resource pooling and redistribution in South African healthcare, the specific context of the country's health landscape must be carefully considered. Although the 2019 NHI bill identifies multiple risk pooling due to the current private medical aid schemes as a barrier to effective resource pooling, a cautious approach is advised in deciding between single and multiple risk pooling models [9]. The Council for Medical Schemes (CMS) suggests that reducing product complexity could benefit vulnerable risk groups by providing better protection, and in such an environment, medical schemes would compete based on efficiency rather than risk selection [20]. This perspective aims to alleviate concerns and could inform the decision on whether to adopt single or multiple risk pooling frameworks in South Africa's health system. Table 3 summarizes the discussion of the study findings and actions to address the challenges identified for NHI healthcare financing.

**Table 3:** summary of the actions to address the challenges identified for national health insurance healthcare financing

Subtheme	Identified gap/s or challenges	Action to address concerns
Funding the NHI (raising revenues to expand and sustain coverage)	Insufficient government finances to support the NHI policies and programmes. Be aware of over-reliance on the private sector.	Adopt an incremental approach to expand coverage. Explore different sources of revenue to raise finances, including allowing the growth of private voluntary health insurance (PVHI). Ensure there is sufficient governance and regulation of costs and quality.
Policies that will ensure more value for the money spent	Less than sufficient investments in PHC in order to deal with emergencies and provide essential services that will address the determinants of health. Supply-side policies do not prioritize service and medicine for benefit package expansion.	Prioritize and ramp up investment in PHC services. Device measures to improve efficiencies in managing costs and invokes strategies with pharmaceutical companies to leverage provider payment systems.
Effective pooling and redistribution of resources to ensure equity and financial protection	Divergent views exist on which approach should be adopted.	Debate and agree on monitoring mechanisms to prevent perverse cross-subsidization. Agree on monitoring mechanisms to ensure that coverage grows.

NHI: national health insurance

The limitation in the study is that it was conducted amongst mainly role players concerned with the healthcare industry and the delivery of healthcare. This might have excluded other role players who might have provided a different context to the views provided. It would have been desirable if the study encompassed a much broader audience as opposed to having been restricted to only healthcare industry participants. The bias of the participants, as well as the interpretation of the data, may produce potential limitations. The uncertainty and varied sentiments that exist in the public domain pertaining to the government's approach to the implementation of the NHI may affect outcomes.

## Conclusion

In summary, the study highlighted broad support for government-led, diversified funding approaches for NHI, a preference for value-driven spending and primary care, and mixed views on resource pooling mechanisms. In addition, there is support for seamless co-existence of the public and private sectors, where the private sector medical aid plays a strong role in offering coverage. The modalities of this arrangement need to consider the unique position of the country´s population health needs and constraints to access to healthcare. While consensus exists on some issues across occupational groups, significant differences remain on others, especially regarding resource pooling and value for money. The moderate correlation between subthemes suggests interconnectedness among the various aspects of healthcare financing under NHI.

### What is known about this topic

- The fiscal space to finance UHC policies and programs is highly constrained since coverage expansion calls for a huge increase in public spending;
- Several approaches to fund universal coverage, including various tax options, pooling of revenues, and cost-containment and management improvement measures, have been widely employed by a number of countries with varying success;
- Finally, it is in those countries where funding efforts were context-specific that sufficient service coverage was attained.

### What this study adds

- Exploration of a seamless co-existence of the public and private sectors presents a golden opportunity to be explored by South Africa to ensure adequate coverage of the population;
- There is a need to carefully explore both the risk and benefit presented by the multiple risk pools because of the private medical aid scheme configuration;
- Service coverage and access will be affected by the unique healthcare landscape in South Africa as a result of the long-standing two-tier system.
